# Geosocial-Networking App Usage Patterns of Gay, Bisexual, and Other Men Who Have Sex With Men: Survey Among Users of Grindr, A Mobile Dating App

**DOI:** 10.2196/publichealth.4353

**Published:** 2015-05-08

**Authors:** William C Goedel, Dustin T Duncan

**Affiliations:** ^1^ School of Medicine Department of Population Health New York University New York, NY United States; ^2^ Global Institute of Public Health New York University New York, NY United States; ^3^ College of Arts and Science Department of Sociology New York University New York, NY United States; ^4^ Center for Drug Use and HIV Research College of Nursing New York University New York, NY United States; ^5^ Population Center New York University New York, NY United States; ^6^ Center for Data Science New York University New York, NY United States

**Keywords:** homosexuality, MSM, men who have sex with men, male, mobile apps, dating apps, mobile phones, HIV, AIDS prevention

## Abstract

**Background:**

Geosocial-networking apps like Grindr have been used increasingly among men who have sex with men (MSM) to meet anonymous partners. These mobile dating apps employ global positioning system technology to facilitate connections with other users based on their current location. These new technologies have generated quicker and easier modes for men who have sex with men to meet potential partners based on attraction and physical proximity.

**Objective:**

The aim of this study is to describe geosocial-networking app use and recent sexual behaviors of MSM in the Atlanta metropolitan statistical area.

**Methods:**

Our sample was recruited from Grindr, the most commonly used of these mobile apps among MSM, using broadcast advertising. Advertisements were displayed over the course of a 72-hour period and participants were directed to a Web-based survey.

**Results:**

In total, 604 men clicked through the advertisement, and 92 users completed the survey. One-third (38.0%) of the men reported using these mobile apps to meet new sexual partners, and one-fifth (18.5%) used them to “kill time” when bored. Men reporting currently being in a relationship were less likely to report using these mobile apps to meet other MSM to date or to find a boyfriend or romantic partner, but more likely to report using these mobile apps to meet other MSM to have sex, *X* ^2^_24_=12.1, *P*=.016. Respondents had current accounts on 3.11 mobile apps (SD 1.84) on average, with Grindr being the most common (100%), followed by Scruff (52.5%), and Jack’d (45.7%). Most men were most active in the late night (40.2%), and on weekdays (64.1%). Each day, on average, men reported opening these mobile apps 8.38 times (SD 8.10) and spent 1.31 hours (SD 1.15) on these mobile apps. The age respondents began using these mobile apps was associated with the age at their first instance of insertive anal sex (r_80_=.527, *P*<.001) and receptive anal sex (r_76_=.527, *P*<.001).

**Conclusions:**

These findings suggest that MSM use multiple mobile apps and spend significant time on them. For these reasons, HIV prevention interventions could be delivered on these mobile apps.

## Introduction

Gay, bisexual, and other men who have sex with men (MSM) represent only 2% of the male population in the United States, yet comprised the majority (63%) of all new human immunodeficiency virus (HIV) infections diagnosed in adults and adolescents in the United States in 2010 [1]. The region commonly referred to as the Deep South (Alabama, Georgia, Louisiana, Mississippi, North Carolina, and South Carolina) is disproportionately affected by the HIV/AIDS epidemic. From 2000 to 2003, the number of newly reported acquired immune deficiency syndrome (AIDS) cases increased by 36.5% in this region, while the number of newly reported AIDS causes increased by only 4.0% in the other states in the Southern United States. (Delaware, Maryland, West Virginia, Virginia, Florida, Arkansas, Tennessee, Kentucky, Texas, and Oklahoma) and by 5.2% in the remaining portion of the country [2]. Although only 37% of the US population resides in the South, about half (49%) of individuals living with HIV in 2010 were diagnosed in the South [3,4]. In 2010, Georgia ranked sixth highest in the nation for the total number of adults and adolescents living with HIV. In 2012, among all HIV infections and cases of AIDS in male adults and adolescents in Georgia, 63% of HIV infections and 76% of cases of AIDS were seen in MSM [5].

The Internet is one of the most popular venues for sexual partner seeking among MSM [6,7]. Sexual partner seeking on the Internet encourages the use of partner selection criteria in profiles and these specifications often include the preferred age, race/ethnicity, and body type of a partner as well as the desired sexual practices of the individual [8-12]. Studies suggest that compared to men who do not seek sex on the Internet, Internet sex-seekers tend to have more frequent anal intercourse, more previously diagnosed sexual transmitted infections (STIs), more sexual exposure to men, greater numbers of sexual partners, and greater numbers of sexual partners known to be HIV-positive [13]. Prior research demonstrates that time spent online looking for casual sexual partners may increase the odds of having anal intercourse without a condom [14].

Geosocial-networking apps (mobile dating apps) like Grindr, Jack’d, and Scruff have been used increasingly among MSM to meet anonymous partners [15]. In 2013, Grindr, the most popular of these apps, reported that it had six million users in 192 different countries around the world with 2.5 million new users added in the previous year [15]. These apps employ global positioning system technology to facilitate connections with other users based on their current location [16] and enable their users to scan for nearby users, chat with them, and meet, sometimes for sexual encounters. These new mobile technologies have generated quicker and easier modes for MSM to meet potential partners based on attraction and physical proximity [17].

Use of these apps is commonplace among MSM. In a sample of 379 MSM in Washington, DC, 63.6% of men reported having used an app to meet a sexual partner in the past year [18]. The use of these apps enables an expansion of an individual’s sexual and social networks. Individuals integrating app-met sexual partners into their social networks were nearly twice as likely to have engaged in anal intercourse without a condom compared to individuals who did not integrate these partners into their social networks as seen in a sample of 295 MSM in Los Angeles [19]. Previous research also found that 75.0% of respondents had a sexual encounter with partners they met on Grindr, and reported significantly higher rates of condom use with partners met on Grindr (59.8%) compared to partners met elsewhere (41.9%) [17,19,20].

Despite the increased popularity of these apps, little is known about the behaviors among their users. As such, the purpose of the current study is to describe the use of these apps and the sexual behaviors of MSM in Atlanta, Georgia as they represent a high-risk group for acquiring HIV. Almost two-thirds (64%) of persons living with HIV in Georgia reside in the Atlanta metropolitan statistical area [5] -an area that included twenty-eight counties and 4.9 million people in 2005. To our knowledge, this is the first study to investigate app use and sexual behaviors of geosocial-networking app-using MSM in Atlanta and in the Southern United States as a whole.

## Methods

### Recruitment

**Figure 1:**
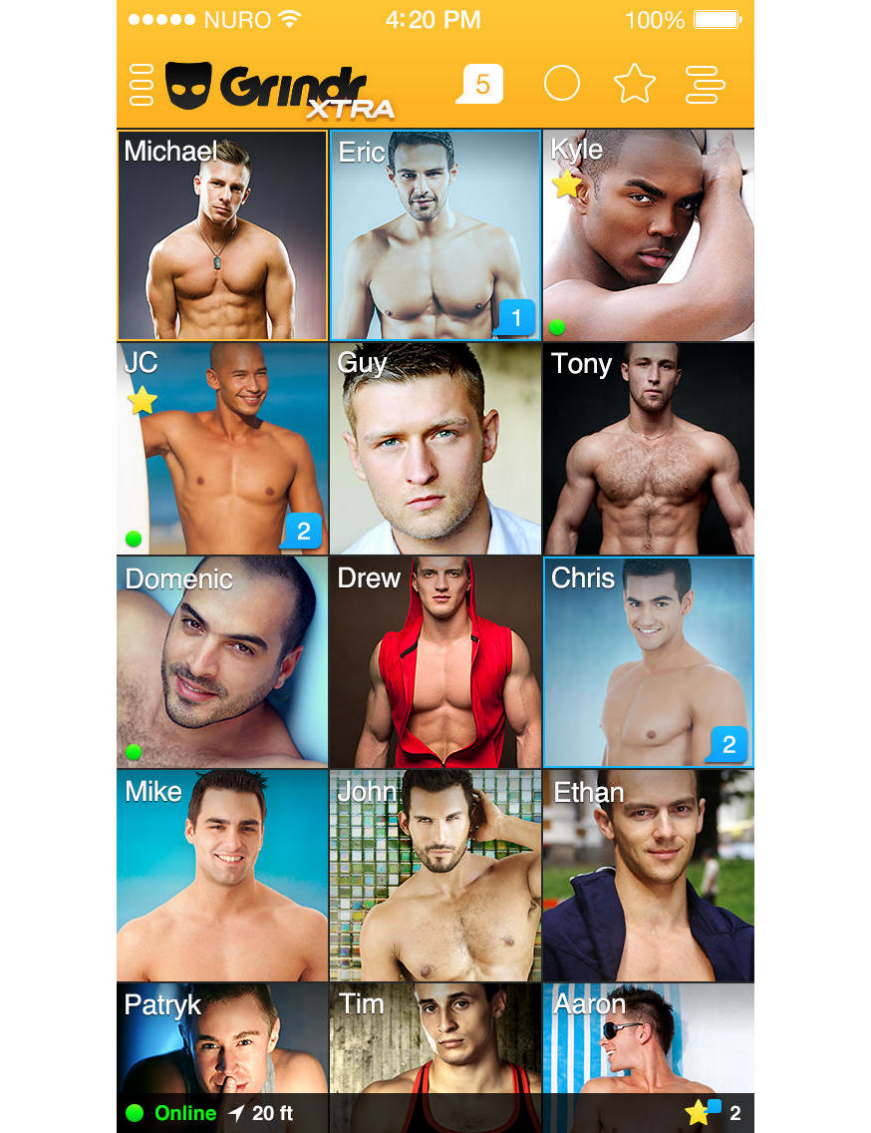
Screenshot of the Grindr user interface.

Figure 1 displays a simulated user interface on Grindr. During a three-day period in January 2015, we advertised on Grindr, utilizing a pop-up advertisement encouraging users to click through to take our survey, a method previously used to recruit MSM into cross-sectional and longitudinal studies of sexual risk taking behavior and substance use [21]. This advertisement was shown the first time that users logged on to the app within a 24-hour period, and was displayed three times during three consecutive 24-hour periods. This advertisement was only shown to Grindr users who logged in to their account in the Atlanta metropolitan area. Participants were alerted that completing the survey entered them for a chance to win one of six $50.00 Amazon gift cards.

The survey took, on average, 25 minutes to complete. All men reported being 18 years of age at survey completion. All procedures were approved by the New York University Committee on Activities Involving Human Subjects. Data were collected anonymously. In total, 604 users clicked through the advertisement and reached the survey, 148 (24.5%) continued to the consent page, 143 (23.7%) provided informed consent, and 92 (15.2%) completed the questionnaire. IP addresses were used to identify potential duplicate entries from the same user over the course of the 72-hour period, but no potential duplicate entries were identified.

### Study Measures

#### Demographic Characteristics

Demographic characteristics were assessed in fourteen items. Age was measured continuously. Sex assigned at birth included male or female. Gender was measured as male, female-to-male transgender, female, male-to-female transgender, and other. Sexual orientation was categorized as gay or homosexual, bisexual, straight or heterosexual, and other. Relationship status was identified as reporting currently being in a relationship or not currently being in a relationship. Race/ethnicity was measured in two items, “Are you Hispanic or Latino?” and “Which of the following best describes your race?” and participants were later categorized as White (non-Hispanic/Latino), Black or African American (non-Hispanic/Latino), Hispanic or Latino (any race), Asian/Pacific Islander, and Multiracial/other based on their responses to these items. National origin was categorized as either being born in the United States or being born outside the United States. Highest educational attainment was categorized as less than twelfth grade, high school or equivalent, some college, trade or vocational training, Associates degree, Bachelor’s degree, Master’s degree, or Doctoral degree. Employment status was categorized as working full time, working part time, not working, currently a student, retired, or unable to work. Individual income in the past year was categorized as under $10,000; $10,000 to $24,999; $25,000 to $39,999; $40,000 to $54,999; $55,000 to $69,999; $70,000 to $84,999; $85,000 to $99,999; and $100,000 or more.

#### Geosocial-Networking App Use Patterns

App use behaviors were assessed in three items. The age at which the respondent started using apps to meet other men was measured continuously. The number of years spent using these apps was calculated for each participant by subtracting the age at which the respondent reported beginning to use these apps from the participant’s self-reported age. Motivation for using these apps was asked, “Which best describes your reason for using these apps?” with five options informed by prior work [17,19,20]: “I want to ‘kill time’ when bored,” “I want to make friends with other gay and bisexual men,” “I want to meet other gay and bisexual men to date,” “I want to find a boyfriend or other romantic partner,” and “I want to meet other gay and bisexual men to have sex with.” While we acknowledge that it is possible for transgender individuals to utilize these apps, we estimate, based on prior work assessing MSM-targeted apps and their use, that individuals using these apps were assigned male at birth and identify as male and are seeking others who were assigned male at birth and identify as male [17]. Respondents were asked to check off which apps they currently had profiles or accounts on from a list including nineteen options: Bender, Boy Ahoy, Distinc.tt, DowneLink, Gay Thug Dating, Grindr, GROWLr, Guy Spy, Hornet, Hunters BBS, Jack’d, Maleforce, MISTER, Planet Romeo, Scruff, Skout, u2nite, u4Bear, and VGL, and a space to indicate others not listed. The number of apps a respondent currently had a profile or account on was calculated for each participant.

Average daily activity was assessed in six items. The time of day an individual was most active on these apps was categorized as early morning (2:31am to 6:30am), morning (6:31am to 12:00pm), afternoon (12:01pm to 5:00pm), evening (5:01pm to 9:30pm), and late night (9:31pm to 2:30am). The part of week an individual was most active on these apps was categorized as weekdays (Monday through Thursday) and weekends (Friday through Sunday). The day of week an individual was most active on these apps included Sunday, Monday, Tuesday, Wednesday, Thursday, Friday, or Saturday. The average number of times an individual opens or logs on was measured continuously in response to: “On average, how many times do you open or log on to these apps each day?” The average number of hours spent on these apps each day was measured continuously in response to: “On average, how many minutes or hours do you spend on these apps each day?” The average number of messages sent each day was measured continuously in response to: “On average, how many messages do you send on these apps each day?”

#### HIV Status and Recent Sexual Behaviors

HIV status was categorized as positive, negative, or unknown/never tested and based on participant self-report. The individual’s age at his first instance of insertive anal intercourse and receptive anal intercourse respectively were measured continuously. Sexual behaviors were assessed in eight items. Participants were asked for the number of partners they had met through apps and engaged in anal intercourse (regardless of position) and in anal intercourse without a condom (regardless of position). Respondents were asked whether or not they engaged in insertive and receptive anal intercourse in the past six months. If insertive or receptive anal intercourse was indicated, the respondent was asked about the number of partners he engaged in the particular behavior with, in the past six months (measured continuously), and the number of partners he engaged in the particular behavior without a condom in the past six months (measured continuously).

#### Partner Characteristics

Information was also collected on sexual partners met using apps and was assessed in three items. Relative age of the majority of sexual partners met using apps was categorized as a lot older (>4 years older), slightly older (2-4 years older), approximately the same age, and younger. Race/ethnicity of the majority of sexual partners met on apps was categorized as White (non-Hispanic/Latino), Black or African American (non-Hispanic/Latino), Hispanic or Latino (any race), and Asian/Pacific Islander. Perceived HIV status of the majority of sexual partners met on apps was categorized as positive, negative, or unknown.

#### Sexual Sensation Seeking Scale

The Sexual Sensation Seeking Scale was used to gauge an individual’s propensity to seek out novel or risky sexual stimulation [22-25]. This ten-item instrument employs a four-point Likert-type response format that has an acceptance internal consistency (Cronbach alpha=.75) in gay men [22,23]. Scores on this scale range from 10 to 40, where higher scores indicate a higher propensity to seek out novel or risky sexual stimulation. In this sample, the instrument displayed moderate internal consistency (Cronbach alpha=.68).

### Statistical Analysis

Statistical Package for the Social Sciences (SPSS) version 21 (SPSS IBM, New York, USA) was used to perform all statistical analysis. Descriptive statistics (eg, means, standard deviations) were calculated for demographic characteristics and behavioral characteristics. Differences in these behavioral characteristics by demographic characteristics were examined using analyses of variance (ANOVA) for associations between continuous behavioral variables and categorical demographic variables, chi-square tests of independence for associations between categorical behavioral variables and categorical demographic variables, and Spearman correlations for associations between continuous behavioral variables and continuous demographic variables. Statistical significance was determined by *P*<.05. No post-hoc statistical analyses were conducted.

## Results

### Sample Characteristics

The demographic characteristics of the sample (N=92) are listed in Table 1. The mean age was 31.73 years old (SD 10.77) and ranged from 18 to 66. A majority of the sample identified as non-Hispanic White (63.0%), while 19.6% of the sample identified as non-Hispanic Black, 9.8% identified as Hispanic or Latino of any racial background, 3.3% identified as Asian or Pacific Islander, and 4.3% identified as multiracial. The vast majority of the sample (93.5%) was born in the United States. The majority of the sample identified their sexual orientation as gay (77.2%) or bisexual (21.7%). More than half of the sample (51.2%) graduated college or completed more graduate education. Nearly three-quarters of the sample (72.8%) was employed either on a full-time or part-time basis.

### Geosocial-Networking App Use Patterns

Table 2 shows the geosocial-networking app use behaviors of the sample. The average age at which respondents began using these apps was 26.61 years old (SD 9.80) and ranged from 14 to 55, and on average, they had been using these apps for 4.83 years (SD 3.50). Over one-third of the men reported using these apps to meet other men for sexual encounters (38.0%), and the second most common reason was using these apps to “kill time” when bored (18.5%), following by using these apps to make friends with other men (17.4%), to find a boyfriend or romantic partner (14.1%), and to meet other gay and bisexual men to date (10.9%). Men reporting currently being in a relationship were less likely than men not currently in a relationship to report using these apps to meet other men who have sex with men to date (0.0% vs 14.7%) or to find a boyfriend or other romantic partner (0.0% vs 19.1%), but more likely to report using these apps to meet other men who have sex with men to have sex (60.9% vs 30.9%), χ^2^_4_=12.1, *P*=.02.

Respondents, on average, reported having current accounts or profiles on 3.11 apps (SD 1.84), with Grindr being the most common (100%), followed by Scruff (52.5%), Jack’d (45.7%), Hornet (21.8%), and GROWLr (18.5%). Most men were active in the evening (34.8%) or late night (40.2%), and on weekdays (64.1%)—compared to early morning (6.5%), morning (8.7%), and afternoon (9.8%) hours, and weekends (35.9%). Being active on weekdays was associated with having a lower individual income in the past year, χ^2^_7_=23.5, *P*=.001, and being currently unemployed, χ^2^_4_=13.9, *P*=.008. Each day, on average, men logged on or opened these apps 8.38 times (SD 8.10), spent 1.31 hours (SD 1.15), and sent 21.03 messages (SD 25.62).

**Table 1:** Sample Demographics.

Demographics		Frequency	Percentage (%)^a^
Sex assigned at birth (Male)		90	97.8
Gender identity (Male)		92	100.0
**Sexual orientation**			
	Gay	71	77.2
	Bisexual	20	21.7
	Other	1	1.1
**Current relationship**			
	Yes	23	25.0
	No	69	75.0
**Race/ethnicity**			
	White (non-Hispanic/Latino)	58	63.0
	Black (non-Hispanic/Latino)	18	19.6
	Hispanic/Latino (any race)	9	9.8
	Asian/Pacific Islander	3	3.3
	Multiracial	4	4.3
**National origin**			
	United States	86	93.5
	Outside United States	6	6.5
**Education**			
	Less than 12th Grade	2	2.2
	High School, or equivalent	11	12.0
	Some college	21	22.8
	Technical or vocational training	4	4.3
	Associates degree	7	7.6
	Bachelors’ degree	33	35.9
	Masters’ degree	11	12.0
	Doctoral degree	3	3.3
**Employment status**			
	Working full time	52	56.5
	Working part time	15	16.3
	Not working	12	13.0
	Student	11	12.0
	Unable to work	2	2.2
**Individual yearly income**			
	Under $10,000	16	17.4
	$10,000 to $24,999	17	18.5
	$25,000 to $39,999	17	18.5
	$40,000 to $54,999	20	21.7
	$55,000 to $69,999	8	8.7
	$70,000 to $84,999	6	6.5
	$85,000 to $99,999	4	4.3
	$100,000 or more	2	2.2

^a^Percentages may not add to 100 in cases of missing data.

**Table 2:** Geosocial-networking app use (categorical variables).

App use			Frequency	Percentage (%)^a^
**Current accounts/profiles**				
	Grindr		92	100.0
	Scruff		48	52.2
	Jack’d		42	45.7
	Hornet		20	21.7
	GROWLr		17	18.5
	Guy Spy		10	10.9
	MISTER		8	8.7
	Boy Ahoy		7	7.6
	Skout		7	7.6
	Bender		4	4.3
	Planet Romeo		3	3.3
	U4BEAR		3	3.3
	VGL		3	3.3
**Most active time of day**				
		Early morning (2:31am to 6:30am)	6	6.5
		Morning (6:31am to 12:00pm)	8	8.7
		Afternoon (12:01pm to 5:00pm)	9	9.8
		Evening (5:01pm to 9:30pm)	32	34.8
		Late night (9:31pm to 2:30am)	37	40.2
**Most active part of week**				
		Weekdays (Monday through Thursday)	59	64.1
		Weekends (Friday through Sunday)	33	35.9
**Most active day of week**				
		Sunday	6	6.7
		Monday	16	17.4
		Tuesday	6	6.7
		Wednesday	12	13.0
		Thursday	8	8.7
		Friday	21	22.8
		Saturday	21	22.8

^a^Percentages may not add to 100 in cases of missing data.

### Recent Sexual Behaviors

Table 3 displays the self-reported HIV statuses and recent sexual behaviors of all respondents in the sample. HIV-negative individuals constituted 84.8% of the sample, while HIV-positive individuals constituted 8.7% of the sample, and 6.5% reported an unknown HIV status or had never been tested. The average age at the individual’s first instance of insertive anal intercourse (IAI) was 20.64 years old (SD 6.90), ranging from 13 to 56, and at first instance of receptive anal intercourse (RAI) was 20.14 years old (SD 7.14), ranging from 12 to 58. The respondents who were sexually active in the last six months, on average, had 4.28 app-met partners (SD 5.68) in the last six months who they engaged in either anal intercourse (irrespective of role as an insertive or receptive partner). Additionally, these respondents had 2.19 app-met partners (SD 4.72) in the last six months who they engaged in anal intercourse (irrespective of role as an insertive or receptive partner) without a condom. HIV-positive respondents, on average, had 8.00 partners who they met on apps and had anal intercourse without a condom in the last six months, while HIV-negative respondents had anal intercourse without a condom in the last six months with 1.68 app-met partners, *F*_2,86_=7.800, *P*=.001.

**Table 3:** HIV status and recent sexual behaviors.

		Frequency	Percentage (%)^a^
**HIV status**			
	Negative	78	84.8
	Positive	8	8.7
	Unknown/never tested	6	6.5
**Engaged in Insertive Anal Intercourse (IAI) in past 6 months**			
	Yes	65	70.7
	No	27	29.3
**Engaged in Receptive Anal Intercourse (RAI) in past 6 months**			
	Yes	54	58.7
	No	38	41.3

^a^Percentages may not add to 100 in cases of missing data.

### Partner Characteristics

Table 4 reports the characteristics of the majority of the respondent’s app-met partners. Most respondents reported meeting partners that were younger than themselves (34.1%) on these apps; however, 27.5% reported meeting partners greater than four years older than themselves, 25.3% reported meeting partners approximately the same age as themselves, and 13.1% reported meeting partners two to four years older than themselves. Asian and Black respondents were more likely to report pairing with partners who are greater than four years older than themselves, while White and Hispanic/Latino respondents were more likely to report pairing with partners who are about the same age as or younger than themselves, χ^2^_12_=28.6, *P*=.005.

**Table 4:** App-met partner characteristics.

Characteristics		Frequency	^a^Percentage (%)
**Relative age**			
	Younger	31	34.1
	Approximately same age	23	25.3
	2-4 Years older	12	13.2
	>4 Years older	25	27.5
**Race/ethnicity**			
	White (non-Hispanic/Latino)	64	69.6
	Black (non-Hispanic/Latino)	15	16.3
	Hispanic/Latino (any race)	10	10.9
	Asian/Pacific Islander	1	1.1
**Perceived HIV status**			
	Negative	77	84.6
	Positive	3	3.3
	Unknown/never tested	11	12.1

^a^Percentages may not add to 100 in cases of missing data.

Most respondents reported a majority of their partners met on these apps being White (69.6%), and 16.3% reported a majority of their app-met partners being Black. Also, 10.9% reported a majority of their app-met partners being Hispanic/Latino, and 1.1% reported a majority of their app-met partners being Asian. White respondents were more likely to report pairing with White partners (82.1%), compared to Hispanic/Latino, Black/African American, and Asian/Pacific Islander partners, χ^2^_12_=45.9, *P*<.001. Most respondents believed a majority of their partners to be HIV-negative (84.6%) or HIV-unknown (12.1%) compared to those believing a majority of their partners to be HIV-positive (3.3%). HIV-negative respondents were more likely to pair with HIV-negative partners (90.9% vs 37.5%) and HIV-positive respondents were more likely to pair with HIV-positive partners (37.5% vs 0.0%), χ^2^_4_=37.8, *P*<.001.

### Sexual Sensation Seeking Scale

The average Sexual Sensation Seeking Scale score was 30.32 (SD 4.52), ranging from 16 to 40. A higher propensity to seek out novel or risky sexual stimulation was positively associated with the number of partners an individual met on apps and had anal intercourse with (*r*_84_=.269, *P*=.012), the number of partners an individual met on apps and had anal intercourse without a condom (*r*_85_=.362, *P*=.001), the number of partners an individual engaged in anal intercourse in the receptive position (*r*_50_=.457, *P*=.001), and the number of partners an individual engaged in anal intercourse in the receptive position without a condom (*r*_48_=.427, *P*=.002).

## Discussion

### Principal Findings

The purpose of the current study was to describe the usage of geosocial-networking apps among a sample of MSM in Atlanta, Georgia on Grindr. To our knowledge, this is the first study to investigate app use and sexual behaviors of geosocial-networking app users in Atlanta and in the Southern United State as a whole. Most men involved in the study currently had an account or profile on more than one app other than Grindr, with the most common being Scruff, Jack’d, Hornet, and GROWLr. In addition, we found that most men were active in the evening (34.8%) or late night (40.2%), and on weekdays (64.1%). Each day, on average, men logged on or opened these apps 8.38 times, spent 1.31 hours, and sent 21.03 messages. Similarly frequent use of these apps was observed in a sample of 195 Grindr users in Los Angeles, where approximately half (49.7%) reported using Grindr more than five times per day [17].

Individuals indicating being in a relationship were less likely to report using these apps for dating or finding a romantic partner, but more likely to use these apps to find other sexual partners. Among gay couples, agreements about sex with outside partners, whether closed (monogamous) or open, are common, and these agreements serve as a framework for the couples’ decisions regarding sexual behaviors that heighten risk of acquiring HIV. Open agreements may permit a variety of acceptable sexual behaviors with outside partners, such as “being safe” (ie, using a condom) with outside partners [26].

In addition, we found that the age respondents began using these apps was associated with the age at their first instance of insertive anal sex and receptive anal sex. Young MSM often rely on organizations, social events, and the Internet to assist in developing their sexual identity, but in cities with limited community-based organizations, they may rely on informal role models, such as older men and individuals on geosocial-networking apps, to learn about cultural norms in MSM [27,28]. However, little research has directly assessed the age an individual begins to use these geosocial-networking apps and associated sexual behaviors. Thus, our study provides a new contribution to the literature. It is possible that earlier access to other MSM in nearby areas provided by these apps may lead to earlier sexual debut. Sexual debut earlier than 16 years old has been associated with more frequent exchange sex, substance use, emotional and psychological problems related to substance use, and a history of suicide attempts, compared to MSM with later sexual debuts [29].

Many prior studies have compared sexual behaviors, HIV/STI testing and diagnoses, and substance use of app users and non-app users. Psychosocial characteristics, including sensation seeking and self-control, have been compared between MSM who meet partners through apps and MSM who meet partners through other methods. However, no significant differences between the two groups were observed [30]. To our knowledge, this is the first investigation assessing sexual sensation seeking specifically as a correlate of sexual behaviors in MSM who meet partners through apps. However, without a sample of MSM who meet partners through other methods to serve as a control, the significance of findings associated with higher propensities to seek sexual sensation is uncertain and worthy of further investigation.

### Study Implications

This investigation on the usage patterns of geosocial-networking apps has substantial implications for utilizing these apps for HIV prevention efforts—as we show that many men use apps for meeting anonymous sexual partners and that the age respondents began using these apps was associated with the age at their first instance of anal intercourse with another man. While many studies have examined the effectiveness of app-based intervention strategies, these are only effective if downloaded and used by the population at risk [31-33]. Due to their wide use by MSM, it may be best to leverage the use of popularly used and established apps like Grindr, Jack’d, and Scruff for HIV prevention and sexual health promotion. One such app, Hornet, has begun its “Know Your Status” campaign, where HIV status and date of most recent test are featured on profiles. Keeping with this wider pattern of apps targeting MSM promoting sexual health, apps could utilize measures of sexual sensation seeking and recent sexual behavior prior to creating a profile on the app for targeted HIV prevention messages. For example, individuals with a higher propensity to seek sexual sensations may have more frequent sexual encounters and need more frequent reminders for HIV testing.

Previous research among MSM found that 64% of the sample found these apps to be acceptable sources of sexual health information [34], which suggests that these existing tools can be effective intervention targets. Understanding when users are most active, as this work presents, for example, may inform optimal timing for broadcast advertisements used for health promotion services, including information regarding new forms of prevention such as pre-exposure prophylaxis (PrEP), and ways to locate nearby free, confidential HIV testing locations. For example, our work suggests that men are most active on these apps on weekday nights, and health promotion messages could appear more frequently at these higher-use times.

### Future Research

Future research into app use patterns could be conducted in MSM in the Deep South and elsewhere. Such investigations should examine app use in MSM from multiple geographies to enable comparison of use by location—which may have implications for targeted intervention strategies. Indeed, such research would benefit from large population-based samples, not just highly select samples of MSM using only one particular app such as Grindr. We are not aware of any other research on other popular apps, such as Scruff and Jack’d. Additionally, differences in use among various apps should be studied cross-sectionally to investigate variations in behavior and demographics based on the use of specific apps. We recognize different geosocial-networking apps may be popular among different subgroups (eg, Jack’d might be more popular among Black MSM).

In addition, longitudinal research studies could be conducted to examine app use patterns overtime. Longitudinal studies should be conducted to better understand the associations between connecting with potential partners via apps and sexual risk behaviors, with a focus on the impact of varying durations and frequencies of use with age, as young MSM have been found to heavily utilize Internet search engines, gay-friendly chat rooms, pornography websites, and apps targeted to MSM to gain information on sexual behavior, identity, and health [35-38].

### Limitations

The results of this study should be considered in light of their limitations. First, our sample is a relatively small sample of MSM who use geosocial-networking app in Atlanta recruited exclusively from Grindr. A substantial percentage of individuals (83.9%) who saw the advertisement and clicked on it did not complete the survey; as a result, the sample is likely to be biased by some degree of self-selection. For this reason, and given the small sample size, these findings may not be representative of the population of Grindr users in Atlanta or non-Grindr app users in Atlanta.

Furthermore, the behaviors in this study were assessed with self-report measures. While there can be some misclassification (eg, inaccurate reporting) in self-report measures, the survey was conducted anonymously by MSM over their mobile phone or tablet computers, so they may be more likely to answer accurately and honestly. Finally, this was a cross-sectional study app use patterns at one point in time. It is possible that MSM can vary their use patterns over time, perhaps due to seasonality and current relationship status. For example, it is possible that men might be more active on apps during the winter months when they may be more likely to be in their homes, and may be inactive on these apps throughout the course of a monogamous relationship.

### Conclusions

Despite these limitations, this study has a number of strengths and provides meaningful insights for HIV prevention among MSM. These findings suggest that MSM have accounts on multiple apps simultaneously and spend significant time on these apps each day. For these reasons, HIV prevention interventions for MSM could be delivered through a wide range of apps with a potentially large reach to a high-risk subset of MSM.

## Abbreviations

AIDS: Acquired Immune Deficiency Syndrome
HIV: Human Immunodeficiency Virus
MSM: men who have sex with men
PrEP: Pre-Exposure Prophylaxis
STIs: Sexually Transmitted Infections
